# Supplementary material for: Effects of Sodium Alginate Infusion on Intramammary Immunity Against Subclinical Mastitis in Dairy Cows
Source: Int J Mol Sci. 2025 Jun 9;26(12):5515. doi: 10.3390/ijms26125515 (PMC12192673; doi:10.3390/ijms26125515)
Supplement: Supplementary file 1 [file ijms-26-05515-s001.zip › Supplementary data Figure Milk composition.pdf]

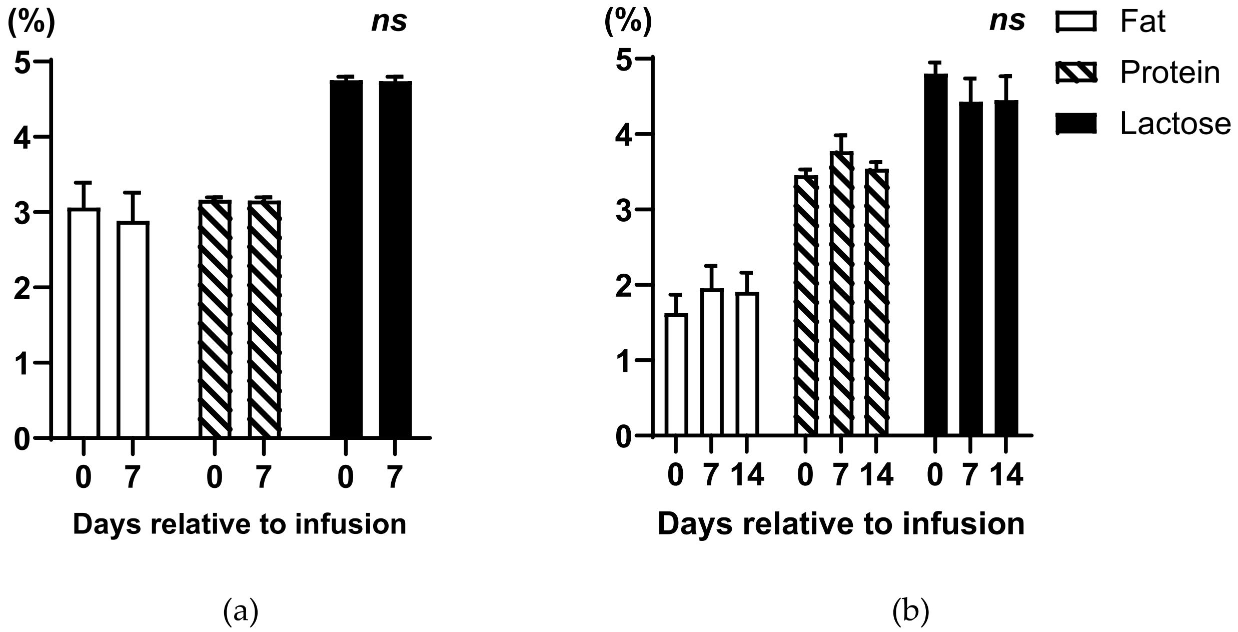

Figure Milk composition before and after sodium alginate treatment in (a) Experiment 1 and (b) Experiment 2. Values are presented as mean ± SEM. ns: not significant.
